# Quantitative EEG Assessment of Dependence-Related Neurophysiological Patterns Using Rule- and Score-Based Modeling in Substance Use Disorders

**DOI:** 10.3390/medicina62030608

**Published:** 2026-03-23

**Authors:** Merve Setenay Gürbüz, Özlem Gül, Eslem Fulya Ekşi, Kültegin Ögel

**Affiliations:** 1 Department of Psychiatry, Nişantaşı University Faculty of Medicine, Istanbul 34398, Türkiye; 2 Department of Psychiatry, Istinye University Faculty of Medicine, Istanbul 34396, Türkiye; ozlemkirtas@hotmail.com; 3 Department of Psychology, Private Moodist Hospital, Istanbul 34660, Türkiye; eksieslem1@gmail.com; 4 Department of Psychology, Faculty of Economics, Administrative and Social Sciences, Kent University, Istanbul 34433, Türkiye; ogelk.net@gmail.com

**Keywords:** electroencephalography, substance use disorder, cortical hyperarousal, dependence likelihood, dependence score, addiction

## Abstract

*Background and Objectives*: Substance use disorders (SUDs) are associated with maladaptive neuroplasticity and chronic dysregulation of cortical arousal. EEG provides a non-invasive tool for quantifying these neurophysiological alterations through spectral power and reactivity indices. Prior research consistently reports elevated beta and diminished alpha activity in SUD, reflecting cortical hyperarousal and reduced inhibitory control. This study sought to identify EEG-based markers of dependence-related neurophysiological alterations by integrating rule-based and score-based models incorporating the theta/beta ratio (TBR), alpha and beta powers, the hyperarousal index, and alpha-blocking measures. *Materials and Methods*: EEG recordings from 47 individuals with SUD were systematically analyzed, focusing on frontal and central cortical regions. Spectral parameters were derived using power spectral density estimation, and composite indices were computed via Python-based signal analysis. A rule-based Dependence Likelihood variable and a continuous Dependence Score (0–1 scale) classified cases as dependence-related (≥0.7), borderline (0.5–0.7), or normal (<0.5). *Results*: Low alpha power and an elevated hyperarousal index (mean = 3.45) characterized most participants. Dependence-related EEG profiles were identified in 87.2% of cases (mean score = 0.86). Alpha blocking remained intact in 46.8% of cases, whereas post-hyperventilation recovery was attenuated in 61.7% of cases. Segmental analysis indicated sustained cortical activation with low TBR (0.37) and elevated beta across all conditions. *Conclusions*: Quantitative EEG analysis revealed consistent hyperarousal and inhibitory deficits in SUD. The combined Dependence Likelihood and Score framework provides an interpretable, reproducible approach for identifying dependence-related EEG signatures and holds promise as a biomarker in addiction neurophysiology.

## 1. Introduction

Substance use disorders (SUDs) represent a complex neurobehavioral disorder underpinned by maladaptive neural plasticity and dysregulation of cortical arousal systems. Electroencephalography (EEG) [1], with its high temporal resolution, provides a powerful non-invasive means of capturing these alterations in oscillatory brain dynamics. Over the past two decades, EEG studies have consistently demonstrated that individuals with SUDs exhibit characteristic deviations in resting-state and task-related spectral activity, particularly within the alpha, beta, and theta frequency bands [2,3,4].

Among the most replicated findings is a pattern of increased beta activity accompanied by reduced alpha power, reflecting cortical hyperarousal and impaired inhibitory control. Sokhadze et al. [2] observed that substance-dependent individuals display excessive beta synchronization and attenuated alpha rhythms, indicative of excitatory dominance within the cortical network. Similarly, Rangaswamy et al. [5] linked heightened beta power to relapse vulnerability and deficient self-regulation. Complementary results from Ceballos et al. [6] and Bel-Bahar et al. [3] further support that this imbalance persists even during resting conditions, suggesting that hyperarousal constitutes a stable neurophysiological trait rather than a transient withdrawal effect.

The theta-to-beta ratio (TBR) has emerged as an integrative marker of cortical activation and attentional regulation. Low TBR values (<1.0) are typically associated with dominance of high-frequency beta oscillations over slower theta waves, a pattern linked to impulsivity and hypervigilance [7,8]. Conversely, normalization or elevation of TBR corresponds to improved inhibitory control and relaxed attentional states [9]. These findings outline a “hyperaroused–disinhibited” EEG phenotype frequently observed in chronic substance users.

In recent years, increasing attention has been directed toward the development of quantitative EEG-based biomarkers and computational frameworks capable of translating complex electrophysiological features into clinically interpretable indices. Such approaches aim to integrate multiple EEG-derived parameters to better characterize neurophysiological dysregulation in psychiatric and addiction-related disorders. Recent reviews and computational studies have highlighted the potential of EEG-derived biomarkers for monitoring neurophysiological alterations and treatment outcomes in substance use disorders [3].

Another sensitive marker of cortical regulation is alpha blocking, the suppression of alpha power upon eye opening [10]. In healthy adults, strong alpha blocking reflects adaptive sensory gating and intact cortical inhibition. In contrast, impaired or absent alpha suppression among dependent individuals points to weakened top-down inhibitory control and reduced cortical reactivity [3]. Supporting evidence from Fathi et al. [11] demonstrates that restoring alpha coherence and reducing excessive beta power through neurofeedback interventions can normalize cortical excitability and enhance abstinence outcomes.

Building upon this body of evidence, we implemented a rule-based quantitative EEG framework to identify dependence-related neurophysiological patterns. Each EEG record was evaluated across multiple spectral parameters, including a Theta/Beta Ratio < 1.0, an Alpha Relative Power < 0.30, a Beta Relative Power > 0.25, a Hyperarousal Index ≥ 2.0, and Alpha Blocking = Impaired. EEGs meeting all these criteria were classified as Dependence-Likely, representing the hyperaroused–disinhibited cortical state consistently described across prior studies. This standardized computational approach enables interpretable and reproducible detection of dependence-linked EEG features and offers a promising neurophysiological framework for precision assessment and monitoring in addiction research.

## 2. Materials and Methods

### 2.1. Ethical Considerations

The study was conducted in accordance with the Declaration of Helsinki and was approved by the Institutional Review Board of Istinye University (protocol code: 24-177, date of approval: 17 October 2024).

### 2.2. Study Design and Participants

This retrospective study included EEG recordings and clinical data from 47 individuals diagnosed with SUDs who were admitted to the addiction treatment unit of Moodist Hospital in 2024. EEG data from patients diagnosed with SUDs who presented to our psychiatry clinic were retrospectively included in the study if their records contained available EEG recordings.

Patients with a history of epilepsy, neurodegenerative disorders, or other neurological or systemic conditions that could potentially affect EEG patterns were excluded. Individuals with major neurological disorders, severe head trauma, or incomplete EEG data were excluded from the analysis. This approach ensured that the analyses specifically reflected electrophysiological alterations associated with substance use.

Demographic and clinical information was obtained from patient medical records and included age, gender, educational and marital status, employment and economic status, physical health, history of psychiatric treatment or medication use, history of suicide attempt, and type and frequency of substance use. Additional variables such as previous addiction treatment and family history of addiction were also recorded.

All participants were diagnosed with SUDs according to the Diagnostic and Statistical Manual of Mental Disorders, Fifth Edition (DSM-5) criteria [12].

### 2.3. EEG Recording Procedure

EEG recordings were obtained using a Neurosoft Neuro-NRS “Neuron-Spectrum” system (Neurosoft, Ivanovo, Russia) with 19 scalp electrodes placed according to the international 10–20 system. Data were stored in European Data Format (.edf) files [13]. All recordings were visually inspected for quality, and channels containing excessive noise or artifacts were excluded prior to analysis. EEG data preprocessing and quantitative analyses were performed using MNE-Python (v0.14) [14].

Artifact inspection was performed through manual visual review of the recordings to identify segments affected by eye movements, muscle activity, or other non-physiological noise. Channels showing persistent contamination were excluded from further analysis to ensure that spectral estimates were derived from artifact-free EEG signals.

Each EEG session consisted of seven standardized segments: background activity, eyes open, eyes closed, hyperventilation, after hyperventilation, flash stimulation, and after flash stimulation [15]. Background, eyes open, and eyes closed segments were successfully obtained in all cases (100%). Hyperventilation and after-hyperventilation segments were present in 95.7% of recordings, while flash stimulation and after-flash stimulation segments were available in 91.5% and 61.7% of cases, respectively (shown in Table 1).

**Figure 1:**
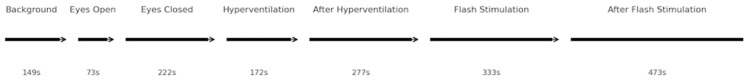
**Segment-based EEG recording protocol and mean durations.** Segment timeline showing seven standardized EEG phases with bar lengths proportional to mean durations (s): 149, 73, 222, 172, 277, 333, and 473, respectively.

The mean durations of EEG segments were as follows: background activity—149 s; eyes open—73 s; eyes closed—222 s; hyperventilation—172 s; after hyperventilation—277 s; flash stimulation—333 s; and after flash stimulation—473 s (shown in Figure 1). Segment boundaries were visually confirmed to ensure consistent epoching across participants. Participants were instructed to remain still, avoid blinking during stimulation, and maintain regular breathing to minimize artifacts throughout the recording.

### 2.4. Channel Selection and Signal Analysis

EEG recordings were obtained from 19 scalp electrodes following the international 10–20 system. For the present analysis, a subset of frontal and central electrodes (FZ–CZ, FP1–F3, FP1–F7, FP2–F4, FP2–F8, F3–C3, F4–C4, C3–P3, C4–P4, CZ–PZ) was selected [15]. This selection was based on prior evidence indicating that frontal regions are critically involved in executive control, attention, and inhibitory processing—functions frequently impaired in addiction—while central regions reflect sensorimotor and cortical arousal activity, providing complementary insights into brain regulation [16].

The raw EEG signals were preprocessed using MNE-Python. After filtering to remove artifacts and noise, power spectral density (PSD) was estimated using Welch’s method as implemented in the MNE-Python library [14]. From the PSD, absolute and relative power values were extracted for the delta (1–4 Hz), theta (4–8 Hz), alpha (8–13 Hz), and beta (13–30 Hz) frequency bands. Additionally, the theta/beta power ratio (TBR) was calculated as an indicator of cortical arousal and attentional control.

**Figure 2:**
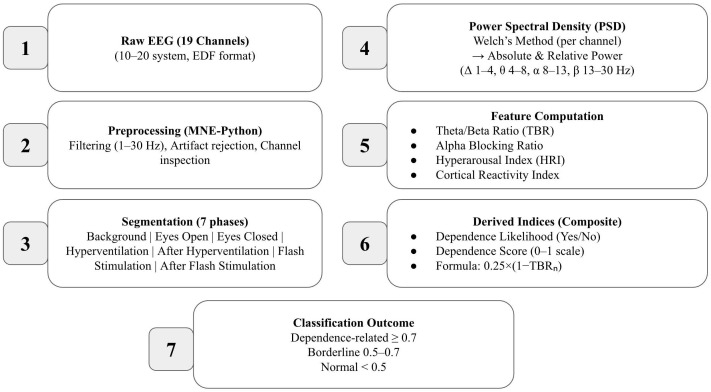
**EEG Data Processing and Feature Extraction Workflow.** A schematic overview of the data processing pipeline. Raw EEG recordings were preprocessed using artifact rejection and band-pass filtering. Power spectral density (PSD) was computed via Welch’s method, followed by derivation of absolute and relative band powers (delta, theta, alpha, beta). Feature extraction included computation of the Theta/Beta Ratio (TBR), Hyperarousal Index (HRI), Alpha Blocking Ratio, and other derived indices used in the Dependence Likelihood and Dependence Score analyses.

To facilitate statistical analysis and reduce feature dimensionality, band powers from all frontal and central channels were averaged separately, resulting in mean frontal and central values for each frequency band and for the TBR metric (shown in Table 2). These aggregated measures were then exported to an Excel dataset for further group-level statistical comparisons. A schematic overview of the complete EEG data processing and feature extraction workflow is illustrated in Figure 2.

### 2.5. Derived EEG Feature Extraction

From the processed EEG data, a set of derived quantitative indices was calculated to capture individual differences in cortical reactivity, arousal balance, and dependence-related neurophysiological patterns. The most relevant measures included the Alpha Blocking Ratio (eyes-closed/eyes-open alpha power), the Theta/Beta Ratio (TBR) as an indicator of attentional control and cortical arousal, the Hyperarousal Index (beta/alpha power ratio), and the Cortical Reactivity Index (CRI) reflecting alpha modulation during stimulation and recovery periods.

In addition, composite variables such as the Dependence Likelihood and the Homeostatic Recovery Score were derived to represent higher-order patterns related to physiological regulation and addiction-related EEG responses. A complete list of the derived EEG variables, their definitions, and calculation methods is provided in Table 3.

### 2.6. Dependence Likelihood Determination and Scoring Framework

To quantify addiction-related neurophysiological patterns, a composite measure termed Dependence Likelihood was established. This index integrates multiple EEG-derived parameters associated with cortical arousal, inhibitory control, and self-regulatory balance, domains frequently altered in SUDs.

The rule-based definition was as follows:

“Yes”: (TBR < 1.0) AND (Background Activity Alpha Relative Mean < 0.30) AND (Background Activity Beta Relative Mean > 0.25) AND (Hyperarousal Index ≥ 2.0) AND (Alpha Blocking Intact = No)

“No”: Otherwise

Each threshold reflects established physiological evidence: a low theta/beta ratio and a low alpha power indicate insufficient cortical inhibition; a high beta power and an elevated hyperarousal index indicate excessive excitatory drive; and absent alpha blocking denotes deficient cortical modulation. Collectively, these parameters represent a state of hyperarousal with impaired inhibitory regulation, characteristic of dependence-related EEG profiles. The selected threshold values were guided by electrophysiological patterns reported in prior EEG studies of substance use disorders, which consistently describe increased beta activity, reduced alpha power, and alterations in theta/beta ratios reflecting cortical hyperarousal and impaired inhibitory regulation.

In addition to the rule-based classification, a continuous Dependence Score was calculated to quantify the multidimensional contribution of EEG features on a normalized scale (0–1). Variable definitions and normalization approaches are presented in Table 4.

The composite score was computed as

Dependence Score = 0.25 × (1 − TBR_norm) + 0.25 × Beta_norm + 0.25 × HRI_norm + 0.15 × (1 − Alpha_norm) + 0.10 × AlphaBlocking_def

This formulation weights inhibitory deficits (low TBR and low alpha) and excitatory dominance (high beta and hyperarousal) more heavily, while incorporating alpha blocking as a modulatory factor.

The weighting coefficients were determined using a physiologically informed heuristic approach rather than statistical model training. Parameters reflecting core mechanisms of cortical excitation and arousal imbalance (TBR, beta power, and the hyperarousal index) were assigned higher weights, while alpha power and alpha blocking were included as complementary markers of inhibitory regulation and cortical reactivity. This design prioritizes interpretability and biological plausibility, while future studies may refine these weights using machine-learning–based optimization in larger datasets.

For interpretability, a combined decision rule was applied:

Dependence-related if Dependence Score ≥ 0.7 or Dependence Likelihood = “Yes”.

Borderline if 0.5 ≤ Dependence Score < 0.7.

Normal pattern if Dependence Score < 0.5.

**Figure 3:**
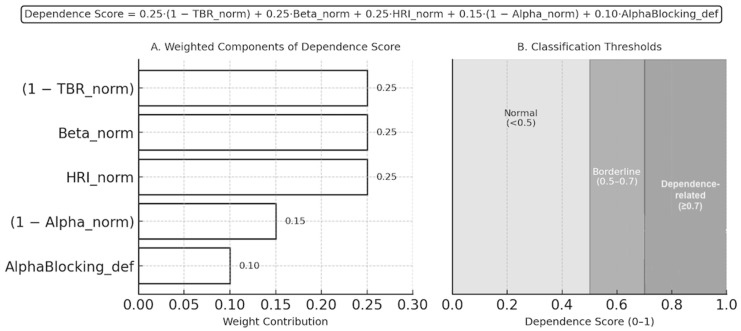
**Dependence Score Computation and Classification Thresholds.** (**A**) Weighted contribution of individual EEG-derived features to the Dependence Score formula: 0.25·(1 − TBR_norm) + 0.25·Beta_norm + 0.25·HRI_norm + 0.15·(1 − Alpha_norm) + 0.10·AlphaBlocking_def. (**B**) Dependence Score classification thresholds: Normal (<0.5), Borderline (0.5–0.7), and Dependence-related (≥0.7). The model integrates inhibitory (alpha, TBR) and excitatory (beta, HRI) features with alpha blocking as a modulatory factor to quantify cortical dysregulation on a normalized 0–1 scale.

This hybrid system allows both rule-based and quantitative assessment, improving classification robustness by aligning categorical thresholds with continuous neurophysiological gradients. (Figure 3) The descriptive results are summarized as Dependence Likelihood Result in Table 5.

### 2.7. Statistical Analyses and Tools

All statistical analyses were performed using Wistats v3.0 (WisdomEra Corp., Istanbul, Türkiye), an integrated Python-based platform running on v2.7.14 for statistical evaluation and machine learning (https://wistats.wisdomera.io, accessed on 1 January 2026). The software incorporates key analytical libraries including SciPy, scikit-learn, and statsmodels, allowing seamless execution of both classical and data-driven analyses within the same computational environment.

Descriptive statistics were calculated for all demographic, clinical, and EEG-derived variables. In addition to descriptive summaries, derived EEG indices such as the Theta/Beta Ratio, the Hyperarousal Index, the Alpha Blocking Ratio, and the composite Dependence Score were examined to characterize the distribution of neurophysiological patterns within the study population. Correlation analyses were conducted to explore relationships between selected EEG-derived measures, particularly between the Theta/Beta Ratio and the Dependence Score, in order to illustrate how inhibitory control and cortical arousal contribute to the composite scoring framework. These analyses were primarily exploratory and aimed to provide descriptive insight into the internal relationships between the quantitative EEG markers used in the proposed model. Comparative analyses between groups or EEG-based categories were performed using appropriate parametric or non-parametric tests according to data distribution, as assessed by Shapiro–Wilk, skewness, and kurtosis measures. Chi-square or Fisher’s exact tests were used for categorical variables, while independent-samples *t*-test, Mann–Whitney U, or Kruskal–Wallis tests were applied for continuous variables. Correlation analyses were conducted using Spearman’s or Pearson’s coefficients as appropriate. All statistical tests were conducted as non-directional (two-tailed) tests, and statistical significance was set at *p* < 0.05.

## 3. Results

### 3.1. Participant Characteristics

A total of 47 patients diagnosed with SUDs were included in the study. Case characteristics are presented in Table 6. The majority of participants were male (76.6%), with a mean age of 31.6 years. Most participants were single (59.6%), had completed university or high school education (53.2% and 27.7%, respectively), and reported medium-to-high economic status (93.6%). Approximately half of the sample were employed (53.2%) and living with their families (46.8%). A psychiatric treatment history was present in 83.0% of cases, and 72.3% reported previous psychiatric drug use. Suicide attempts were reported by 6.4%. Family history of addiction was found in 12.8% of participants. The most common addictive substances were alcohol (48.9%), cannabis (42.6%), and cocaine (23.4%); poly-substance use (≥2 substances) was observed in 42.5% of cases.

### 3.2. Derived EEG Variables

**Figure 4:**
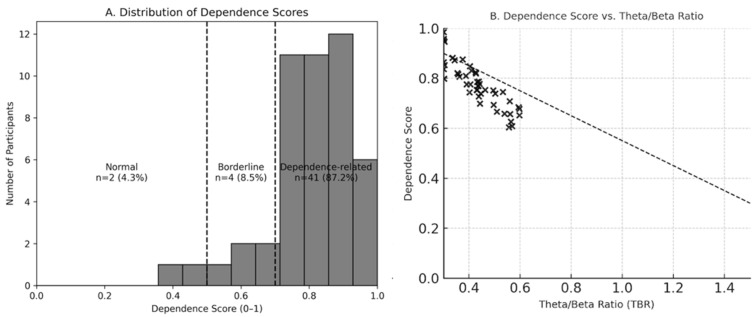
**Distribution and Correlation of Dependence Scores.** (**A**) Histogram showing the distribution of Dependence Scores across all participants, with classification thresholds (Normal < 0.5, Borderline 0.5–0.7, Dependence-related ≥ 0.7) indicated by dashed lines. (**B**) Scatter plot illustrating the inverse relationship between Theta/Beta Ratio (TBR) and Dependence Score, reflecting the link between reduced inhibitory control and elevated cortical arousal in substance use disorder. The dashed line represents a schematic negative trend illustrating the inverse association between TBR and Dependence Score.

Quantitative EEG analysis revealed that all participants demonstrated low alpha power status (100%) and high hyperarousal index values (mean = 3.45). The dependence-related EEG pattern was observed in 87.2% of cases, while 8.5% exhibited borderline patterns and 4.3% showed normal findings. The mean dependence score was 0.86, indicating pronounced cortical arousal and imbalance between alpha and beta activity (Figure 4).

Regarding specific EEG indices, 53.2% of patients showed a hyperaroused dependence pattern, whereas 46.8% were classified as balanced. The alpha blocking ratio averaged 1.60, and 46.8% of participants retained intact alpha blocking. After-hyperventilation alpha recovery was low in 61.7%, and after-flash alpha rebound was absent in 70.2%.

### 3.3. Segment-Based Spectral Power Analysis

**Figure 5:**
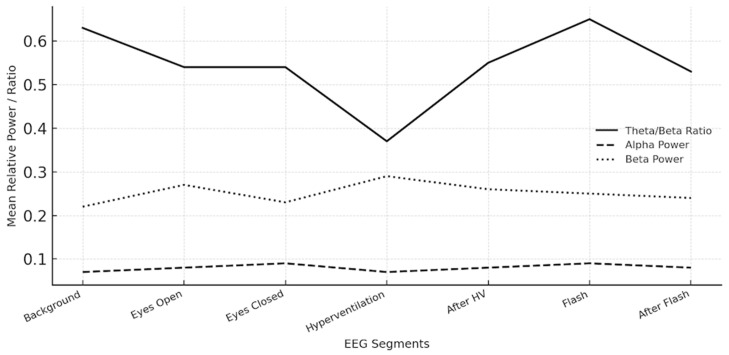
**Mean Spectral Power Distributions Across EEG Segments.** Group-level mean values of the Theta/Beta Ratio, Alpha Relative Power, and Beta Relative Power across seven standardized EEG segments: background, eyes open, eyes closed, hyperventilation, after hyperventilation, flash, and after flash stimulation. The plot highlights persistently low alpha power and elevated beta activity across all conditions, indicating sustained cortical hyperarousal.

PSD analyses calculated by Welch’s method from frontal and central electrodes demonstrated the following mean theta/beta ratios across conditions: background 0.63, eyes open 0.54, eyes closed 0.54, hyperventilation 0.37, after hyperventilation 0.55, flash stimulation 0.65, and after flash stimulation 0.53. Alpha relative power remained consistently low (0.07–0.09) across all conditions, while beta relative power was elevated (0.22–0.29), consistent with a hyperarousal profile. Gamma and delta relative powers were generally low (0.02–0.15). These findings reflect sustained cortical activation and reduced inhibitory regulation during both baseline and stimulation segments (Figure 5).

Collectively, EEG findings in patients with SUDs indicate a predominant hyperarousal pattern, characterized by decreased alpha activity, elevated beta power, and low theta/beta ratios during hyperventilation. The quantitative indices support the presence of dependence-related cortical dysregulation, consistent with neurophysiological models of addictive behavior (shown in Table 5).

## 4. Discussion

The present study identified a consistent hyperaroused–disinhibited cortical profile among individuals with SUDs, characterized by low alpha power, elevated beta activity, and reduced theta/beta ratios across EEG segments. Quantitative indices revealed that 87.2% of participants exhibited dependence-related EEG patterns, while the mean Dependence Score (0.86) indicated pronounced cortical excitation and diminished inhibitory regulation. These findings align with the theoretical framework of addiction as a state of sustained cortical hyperarousal and impaired top-down control, supporting the potential of EEG-based biomarkers for objective assessment of dependence severity.

Prior EEG studies on addiction have consistently reported increased beta synchronization and reduced alpha rhythms, reflecting excessive cortical excitability and deficient inhibitory control [2,3,6]. Similarly, the TBR has emerged as a sensitive marker of attentional regulation and arousal balance, where low TBR values (<1.0) indicate impulsivity and hypervigilance [7,8]. The current findings replicate and extend these results by demonstrating that such spectral imbalances persist across multiple stimulation conditions, including hyperventilation and flash segments, suggesting a trait-like neurophysiological signature rather than a transient state effect. This pattern may reflect a more global disturbance of cortical regulation rather than stimulus-specific dysregulation, potentially contributing to the broader behavioral and interpersonal difficulties frequently observed in individuals with substance use disorders.

The present findings regarding the theta/beta ratio (TBR) align with recent evidence emphasizing its role as a sensitive marker of cortical arousal and attentional regulation. A study demonstrated that lower TBR values are associated with increased cortical excitation, impulsivity, and hypervigilance, reflecting a dominance of fast-frequency beta oscillations over slower theta rhythms [17]. This interpretation supports the current observation that participants exhibiting reduced TBR values also presented elevated beta power and diminished alpha activity, consistent with a hyperaroused–disinhibited cortical profile. Together, these findings reinforce the view that diminished TBR may represent a stable electrophysiological correlate of impaired inhibitory control in substance use disorders.

Unlike prior research that analyzed single spectral parameters or opaque multivariate models, this study introduces a transparent, mathematically defined composite score integrating inhibitory (TBR, alpha) and excitatory (beta, hyperarousal) features with a modulatory alpha-blocking term. The Dependence Score formula, defined as

(0.25 × (1−TBR_norm) + 0.25 × Beta_norm + 0.25 × HRI_norm + 0.15 × (1−Alpha_norm) + 0.10 × AlphaBlocking_def)

enables interpretable quantification of cortical dysregulation on a 0–1 scale, with explicit thresholds for clinical interpretability. To our knowledge, comparable weighted and normalized scoring systems have not been explicitly described in addiction EEG literature [3,6].

The combined rule-based and continuous scoring approach offers multiple advantages:

Interpretability:

Clear weighting and thresholding allow replication and clinical translation.

Granularity:

Borderline scoring (0.5–0.7) captures intermediate regulation states often missed by binary classification.

Reproducibility:

The explicit formula enables comparison across datasets and laboratories.

This framework thus bridges the gap between traditional spectral EEG analysis and potential quantitative EEG markers, potentially supporting relapse risk assessment and personalized treatment monitoring. From a clinical perspective, the Dependence Score may offer a practical tool for monitoring neurophysiological changes during addiction treatment. For example, reductions in the score over time could reflect improvements in cortical inhibitory regulation or normalization of hyperarousal patterns during abstinence or therapy. Conversely, persistently elevated scores may indicate ongoing cortical dysregulation and a potentially increased vulnerability to relapse. In research settings, the scoring framework may also facilitate standardized stratification of participants according to neurophysiological dependence profiles, enabling more precise evaluation of treatment interventions such as neurofeedback, pharmacological therapy, or behavioral rehabilitation programs. Although the present findings are exploratory, these potential applications highlight how quantitative EEG metrics may complement clinical assessments in addiction research.

Recent studies have also explored broader quantitative EEG strategies for extracting clinically meaningful information from brain dynamics beyond single-band spectral comparisons. Across both psychiatric and neurological disorders, EEG-derived features such as spectral power profiles, band ratios, microstate dynamics, connectivity measures, entropy-based indices, and other non-classical markers have been increasingly investigated for diagnosis, prognosis, treatment stratification, and longitudinal monitoring [18,19,20,21,22].

Recent translational and methodological studies have emphasized that the clinical value of EEG is likely to increase when electrophysiological features are standardized, combined within interpretable frameworks, and evaluated in clearly defined clinical contexts rather than considered in isolation [23,24].

In SUDs specifically, emerging work has highlighted the potential relevance of extending quantitative EEG beyond conventional absolute or relative power measures, incorporating more integrative and functionally meaningful electrophysiological indices [25,26]. At the same time, evidence from broader EEG biomarker research in conditions such as neurodegenerative disorders, epilepsy, and critical care settings further supports the utility of multi-feature and clinically oriented analytical approaches [20,27]. Within this perspective, the present Dependence Score should be viewed as an exploratory attempt to integrate multiple physiologically relevant EEG dimensions into a transparent and interpretable composite framework, which may facilitate structured characterization of dependence-related cortical dysregulation and guide future validation studies.

In addition, recent studies have further demonstrated that EEG-derived spectral and time–frequency features are closely associated with cognitive performance and cortical modulation processes, and may provide clinically meaningful information for both assessment and neuromodulatory interventions [28,29].

In parallel, recent advances in electrophysiological signal processing and network-level analysis have further expanded the scope of quantitative EEG beyond traditional spectral metrics. Contemporary studies have emphasized the importance of time–frequency decomposition methods, waveform characteristics, and oscillatory dynamics for capturing physiologically meaningful brain activity patterns [30,31,32,33].

Moreover, growing evidence highlights the relevance of EEG-derived biomarkers related to cognitive control, functional connectivity, and large-scale network organization in neuropsychiatric conditions, suggesting that such multidimensional features may provide additional clinically relevant information beyond conventional power-based measures [34,35,36,37].

In this context, network-oriented and connectivity-based EEG approaches have been increasingly proposed as complementary tools for understanding complex brain dynamics and for developing clinically applicable biomarkers [38,39].

In addition to monitoring neurophysiological changes during treatment, quantitative EEG metrics may also have potential relevance for guiding neuromodulatory interventions in SUDs. Neurofeedback-based therapies have been increasingly explored as non-pharmacological strategies aimed at modulating abnormal cortical activity patterns associated with addiction. A recent systematic review by Fathi et al. [11] reported that both EEG- and fMRI-based neurofeedback interventions have shown promising results in reducing craving and improving psychological outcomes in individuals with SUDs. Notably, many EEG neurofeedback protocols specifically target oscillatory patterns involving increased beta activity and reduced alpha power, which are closely related to the cortical hyperarousal mechanisms examined in the present study. In this context, composite quantitative indices such as the Dependence Score may potentially provide structured metrics for characterizing baseline neurophysiological profiles and for monitoring treatment-related changes in cortical oscillatory dynamics during rehabilitation programs.

While the weighting scheme was guided by empirical rationale, it remains heuristic and should be validated in larger, independent cohorts. Another limitation of the present study is the absence of a healthy control group. As a result, the discriminative performance of the proposed EEG markers and the Dependence Score in distinguishing individuals with substance use disorders from non-SUD populations could not be directly evaluated. Future studies including healthy controls and other psychiatric comparison groups will be necessary to assess the diagnostic specificity and classification performance of this framework and to determine whether the identified EEG patterns represent biomarkers specific to substance dependence or more general markers of cortical dysregulation. The sample size was modest and restricted to patients with established SUD; future studies should examine whether the same scoring framework discriminates between active users, abstinent individuals who maintain abstinence, individuals who subsequently relapse, and healthy controls. Moreover, optimization of weights via machine-learning algorithms and evaluation of test–retest reliability would strengthen the robustness of this model. In addition, the relatively small sample size (*n* = 47) represents an important limitation that may affect the stability and generalizability of the proposed framework. Because the present study introduces a rule- and physiology-based scoring system rather than a statistically trained predictive model, cross-validation or resampling procedures were not applied. Future studies including larger and independent cohorts will be necessary to evaluate the robustness, reproducibility, and classification performance of the Dependence Score using formal validation strategies such as cross-validation or external dataset testing.

In addition, several potential confounding factors should be considered when interpreting the EEG findings. The study sample was derived from a convenience sample within a single health system, which may introduce selection bias and limit the generalizability of the findings to broader populations. Participants represented a clinically heterogeneous population with variability in substance type, patterns of use, and duration of dependence. Differences in substance exposure, polysubstance use, and treatment history may influence cortical oscillatory dynamics and contribute to interindividual variability in EEG measures. Furthermore, psychiatric comorbidities and the use of psychotropic medications—both common in individuals with substance use disorders—may affect EEG spectral activity and cortical arousal patterns. Because these variables were not systematically controlled in the present analysis, their potential influence on the observed EEG features cannot be fully excluded.

## 5. Conclusions

This study proposes a transparent rule-based computational framework for integrating multiple EEG-derived indices associated with cortical arousal and inhibitory regulation in individuals with SUD. The observed patterns—characterized by increased beta activity, reduced alpha power, and alterations in the theta/beta ratio—are consistent with previously reported electrophysiological features of cortical hyperarousal and impaired inhibitory control in addiction.

The proposed Dependence Score provides an interpretable method for combining these neurophysiological signals into a composite metric that may facilitate the quantitative characterization of dependence-related EEG patterns. However, the present findings should be interpreted within the exploratory scope of this study. The absence of a control group, the modest sample size, and the lack of external validation limit the generalizability of the proposed framework.

Future studies including larger and independent cohorts, as well as comparisons with healthy and clinical control populations, will be necessary to validate the robustness, specificity, and potential clinical relevance of the proposed scoring approach.

## Figures and Tables

**Table 1:** Presence of EEG Recording Segments in Patient Data.

	EEG of Cases %
Background Activity Segment	
yes	47 (100.0%)
Open Eyes Segment	
yes	47 (100.0%)
Close Eyes Segment	
yes	47 (100.0%)
Hyperventilation Segment	
no	2 (4.3%)
yes	45 (95.7%)
After Hyperventilation Segment	
no	2 (4.3%)
yes	45 (95.7%)
Flash Stimulation Segment	
no	4 (8.5%)
yes	43 (91.5%)
After Flash Stimulation Segment	
no	18 (38.3%)
yes	29 (61.7%)

**Table 2:** Mean Absolute and Relative Power Spectral Density Values Calculated by Welch’s Method from Segment-Based EEG Analysis in Frontal and Central Regions.

Band	Calculated Variable	Mean
Theta/Beta Ratio	After Flash Stimulation	0.53
	After Hyperventilation	0.55
	Background Activity	0.63
	Eyes Closed	0.54
	Eyes Open	0.54
	Flash Stimulation	0.65
	Hyperventilation	0.37
Alpha	After Flash Stimulation Alpha Absolute	0
	After Flash Stimulation Alpha Relative	0.07
	After Hyperventilation Alpha Absolute	5.96 × 10^−12^
	After Hyperventilation Alpha Relative	0.08
	Background Activity Alpha Absolute	1.34 × 10^−11^
	Background Activity Alpha Relative	0.08
	Eyes Closed Alpha Absolute	5.21 × 10^−12^
	Eyes Closed Alpha Relative	0.09
	Eyes Open Alpha Absolute	0
	Eyes Open Alpha Relative	0.07
	Flash Stimulation Alpha Absolute	6.30 × 10^−12^
	Flash Stimulation Alpha Relative	0.08
	Hyperventilation Alpha Absolute	4.99 × 10^−12^
	Hyperventilation Alpha Relative	0.09
Beta	After Flash Stimulation Beta Absolute	0
	After Flash Stimulation Beta Relative	0.2
	After Hyperventilation Beta Absolute	2.10 × 10^−11^
	After Hyperventilation Beta Relative	0.26
	Background Activity Beta Absolute	4.11 × 10^−11^
	Background Activity Beta Relative	0.22
	Eyes Closed Beta Absolute	1.43 × 10^−11^
	Eyes Closed Beta Relative	0.26
	Eyes Open Beta Absolute	0
	Eyes Open Beta Relative	0.22
	Flash Stimulation Beta Absolute	1.76 × 10^−11^
	Flash Stimulation Beta Relative	0.24
	Hyperventilation Beta Absolute	1.43 × 10^−11^
	Hyperventilation Beta Relative	0.29
Gamma	After Flash Stimulation Gamma Absolute	0
	After Flash Stimulation Gamma Relative	0.02
	After Hyperventilation Gamma Absolute	0
	After Hyperventilation Gamma Relative	0.03
	Background Activity Gamma Absolute	0
	Background Activity Gamma Relative	0.02
	Eyes Closed Gamma Absolute	0
	Eyes Closed Gamma Relative	0.02
	Eyes Open Gamma Absolute	0
	Eyes Open Gamma Relative	0.02
	Flash Stimulation Gamma Absolute	0
	Flash Stimulation Gamma Relative	0.03
	Hyperventilation Gamma Absolute	0
	Hyperventilation Gamma Relative	0.02
Theta	After Flash Stimulation Theta Absolute	0
	After Flash Stimulation Theta Relative	0.06
	After Hyperventilation Theta Absolute	1.04 × 10^−11^
	After Hyperventilation Theta Relative	0.07
	Background Activity Theta Absolute	2.51 × 10^−11^
	Background Activity Theta Relative	0.08
	Eyes Closed Theta Absolute	8.03 × 10^−12^
	Eyes Closed Theta Relative	0.07
	Eyes Open Theta Absolute	0
	Eyes Open Theta Relative	0.07
	Flash Stimulation Theta Absolute	1.88 × 10^−11^
	Flash Stimulation Theta Relative	0.07
	Hyperventilation Theta Absolute	1.38 × 10^−11^
	Hyperventilation Theta Relative	0.07
Delta	After Flash Stimulation Delta Absolute	4.66 × 10^−12^
	After Flash Stimulation Delta Relative	0.23
	After Hyperventilation Delta Absolute	1.38 × 10^−11^
	After Hyperventilation Delta Relative	0.13
	Background Activity Delta Absolute	0
	Background Activity Delta Relative	0.14
	Eyes Closed Delta Absolute	0
	Eyes Closed Delta Relative	0.11
	Eyes Open Delta Absolute	0
	Eyes Open Delta Relative	0.15
	Flash Stimulation Delta Absolute	2.37 × 10^−12^
	Flash Stimulation Delta Relative	0.13
	Hyperventilation Delta Absolute	0
	Hyperventilation Delta Relative	0.1

**Table 3:** Derived EEG Variables Used for Quantitative Feature Extraction and Classification.

Derived EEG Indices	Variable Type	Definition/Calculation
After Flash Stimulation Alpha Rebound	Categorical	“Yes” if After Flash Alpha Relative Mean > Flash Alpha Relative Mean; “No” otherwise
After Hyperventilation Alpha Recovery	Categorical	“Low” if After HV Alpha < HV Alpha; “Normal” if After HV Alpha > HV Alpha
After Hyperventilation Delta Increase	Categorical	“Yes” if After HV Delta > HV Delta; “No” otherwise
Alpha Blocking Ratio	Continuous	Eyes Closed Alpha Relative Mean/Eyes Open Alpha Relative Mean
Alpha Blocking Intact	Categorical	“Yes” if Alpha Blocking Ratio ≥ 1.3; “No” if <1.3
Alpha Power Status	Categorical	“Low” if Background Alpha Relative Mean < 0.25; “Normal” if ≥0.25
Beta Power Status	Categorical	“High” if Background Beta Relative Mean > 0.20; otherwise “Normal”
Cortical Reactivity Index	Continuous	Mean of [(Eyes Closed Alpha − Eyes Open Alpha) + (After Flash Alpha − Flash Alpha)]/2
Cortical Reactivity Index Category	Categorical	“Low” (<0), “Normal” (0–0.05), “High” (>0.05)
Dependence Likelihood	Categorical	“Yes” if (TBR < 1.0) & (Alpha < 0.30) & (Beta > 0.25) & (Hyperarousal ≥ 2.0) & (Alpha Blocking = No); “No” otherwise
Dependence Score	Continuous	Composite index: 0.25 × (1 − TBR_norm) + 0.25 × Beta_norm + 0.25 × HRI_norm + 0.15 × (1 − Alpha_norm) + 0.10 × AlphaBlock_def (0–1 range)
Dependence Likelihood Result	Categorical	Descriptive interpretation: dependence-related, borderline, or normal pattern
Dependence Pattern	Categorical	“Hyperaroused” if Alpha Low + Beta High; “Disinhibited” if TBR Low + Beta High; otherwise “Balanced”
Flash Stimulation Beta Reactivity	Categorical	“Yes” if Flash Beta Relative Mean > Background Beta Relative Mean; “No” otherwise
Homeostatic Recovery Category	Categorical	“Low” if Score < 0.8; “Normal” if 0.8–1.2; “High” if >1.2
Homeostatic Recovery Score	Continuous	After Hyperventilation Alpha Relative Mean/Hyperventilation Alpha Relative Mean
Hyperarousal Index	Continuous	Background Beta Relative Mean/Background Alpha Relative Mean
Hyperarousal Index Category	Categorical	“Low” (<1.0), “Normal” (1.0–2.0), “High” (>2.0)
Theta/Beta Ratio Category	Categorical	“Low” if <1.0; “Normal” if 1.0–4.0; “High” if >4.0

**Table 4:** Normalization and Feature Scaling of EEG-Derived Variables Used in Dependence Score Calculation.

Variable	Normalization Formula	Normalization Range	Rationale	Effect on Dependence Score
TBR	tbr_norm = min (tbr/4.0, 1.0)	0–1 (truncated at 1.0)	TBR values between 1.0 and 4.0 represent normal cortical activation; dividing by 4 scales values to [0–1]. Values > 4.0 capped to prevent overweighting.	Used as (1 − TBR_norm): low TBR (excess beta) increases dependence score.
Alpha Relative Power	alpha_norm = min (alpha/0.30, 1.0)	0–1	Alpha < 0.30 reflects reduced cortical inhibition and hyperarousal. Normalization ensures proportional scaling to the physiological range.	Used as (1 − Alpha_norm): lower alpha increases dependence score.
Beta Relative Power	beta_norm = min (beta/0.25, 1.0)	0–1	Beta > 0.25 indicates excessive cortical excitation; normalization anchors the hyperarousal threshold.	Higher beta_norm increases dependence score.
HRI	HRI_norm = min (HRI/3.0, 1.0)	0–1	Typical HRI (Beta/Alpha) values around 1–3; dividing by 3 limits extreme excitatory dominance.	Higher HRI_norm increases dependence score.
Alpha Blocking Deficit	AlphaBlocking_def = 1 if AlphaBlocking = “No” else 0	Binary (0 or 1)	Absence of alpha blocking reflects impaired sensory gating and cortical modulation.	Adds 0.10 weight when alpha blocking is absent, increasing dependence score.

Each parameter is first scaled to a 0–1 range, ensuring comparability and preventing any single feature from dominating the composite index. The “min()” truncation limits the impact of extreme values and stabilizes interindividual variability. Parameters indicating inhibitory deficits or excitatory dominance (low TBR, low alpha, high beta, high HRI, absent alpha blocking) contribute positively to the final Dependence Score, consistent with the hyperarousal–disinhibition model of substance dependence. TBR: Theta/Beta Ratio, HRI: Hyperarousal Index.

**Table 5:** Derived EEG Variables and Computational Definitions in Patients with Substance Use Disorder.

	Substance Use Disorder Mean/%
After Hyperventilation Delta Increase	
No	26 (55.3%)
Yes	21 (44.7%)
Alpha Blocking Intact	
No	25 (53.2%)
Yes	22 (46.8%)
Flash Stimulation Beta Reactivity	
No	24 (51.1%)
Yes	23 (48.9%)
Theta/Beta Ratio Category	
Low	39 (83.0%)
Normal	8 (17.0%)
Homeostatic Recovery Score	0.78
After Flash Stimulation Alpha Rebound	
No	33 (70.2%)
Yes	14 (29.8%)
Cortical Reactivity Index Category	
High	5 (10.6%)
Low	23 (48.9%)
Normal	19 (40.4%)
Homeostatic Recovery Category	
High	1 (2.1%)
Low	20 (42.6%)
Normal	26 (55.3%)
Dependence Pattern Label	
Balanced	22 (46.8%)
Hyperaroused	25 (53.2%)
Alpha Power Status	
Low	47 (100.0%)
Dependence Likelihood Result	
borderline	4 (8.5%)
dependence-related	41 (87.2%)
normal pattern	2 (4.3%)
Cortical Reactivity Index	−0.0044
Dependence Score	0.86
Beta Power Status	
High	25 (53.2%)
Normal	22 (46.8%)
After Hyperventilation Alpha Recovery	
Low	29 (61.7%)
Normal	18 (38.3%)
Alpha Blocking Ratio	1.60
Hyperarousal Index	3.45
Hyperarousal Index Category	
High	33 (70.2%)
Low	4 (8.5%)
Normal	10 (21.3%)
Dependence Likelihood	
No	41 (87.2%)
Yes	6 (12.8%)

**Table 6:** Case Characteristics and Comparative Results of Variables in Substance Use Disorder Cases.

	Substance Use Disorder Mean/%
*n*	47 (100%)
Gender	
female	11 (23.4%)
male	36 (76.6%)
Age	31.6
Marital Status	
married	19 (40.4%)
single	28 (59.6%)
Educational Status	
High School	13 (27.7%)
Middle School	9 (19.1%)
University	25 (53.2%)
Economic Status	
High	44 (93.6%)
Medium	3 (6.4%)
Employed?	
no	22 (46.8%)
yes	25 (53.2%)
Lives with Family	
no	25 (53.2%)
yes	22 (46.8%)
Lives with Spouse	
no	29 (61.7%)
yes	18 (38.3%)
Lives Alone	
no	41 (87.2%)
yes	6 (12.8%)
Physical Health	
good	47 (100.0%)
History of Psychiatric Treatment	
no	8 (17.0%)
yes	39 (83.0%)
History of Psychiatric Drug Usage	
no	13 (27.7%)
yes	34 (72.3%)
Suicide Attempt	
no	44 (93.6%)
yes	3 (6.4%)
Addiction Type Count	
1	27 (57.4%)
2	15 (31.9%)
3	4 (8.5%)
4	1 (2.1%)
History of Addiction Treatment	
no	12 (25.5%)
yes	35 (74.5%)
Family History of Addiction	
no	41 (87.2%)
yes	6 (12.8%)
Addiction In Mother	
no	46 (97.9%)
yes	1 (2.1%)
Addiction In Father	
no	44 (93.6%)
yes	3 (6.4%)
Addiction In Sister	
yes	1 (2.1%)
no	46 (97.9%)
Addiction In Brother	
no	45 (95.7%)
yes	2 (4.3%)
Alcohol	
no	24 (51.1%)
yes	23 (48.9%)
Alcohol Every X Days	
1	21 (91.3%)
2	2 (8.7%)
Heroin	
no	46 (97.9%)
yes	1 (2.1%)
Heroin Every X Days	
1	1 (100.0%)
Gambling	
no	44 (93.6%)
yes	3 (6.4%)
Gambling Every X Days	
1	3 (100.0%)
Cocaine	
no	36 (76.6%)
yes	11 (23.4%)
Cocaine Every X Days	
1	8 (72.7%)
3	2 (18.2%)
30	1 (9.1%)
Cannabis	
no	27 (57.4%)
yes	20 (42.6%)
Cannabis every X days	19 (95.0%) 1 (5.0%)
1	
2	
Met	
no	43 (91.5%)
yes	4 (8.5%)
Met every x days	
1	4 (100.0%)
Other substance	
no	36 (76.6%)
yes	11 (23.4%)
Other substance every x days	10 (90.9%) 1 (9.1%)
1	
3	

*n*: Number of cases.

## Data Availability

The data produced and examined in the present study are available through the Istinye University Dataset Sharing Platform. De-identified clinical datasets may be accessed via the following link: https://dataset.istinye.edu.tr/dataset?did=64 (accessed on 20 February 2026). All records were anonymized in full compliance with applicable ethical standards. Data access is granted exclusively for research use within a controlled-access framework, in accordance with the platform’s established data-sharing and licensing policies. The shared dataset contains de-identified, processed variables suitable for analysis rather than raw clinical records to ensure compliance with ethical and data-protection standards.

## Abbreviations

The following abbreviations are used in this manuscript:

EEG	Electroencephalography
SUD	Substance Use Disorder
TBR	Theta/Beta Ratio
HRI	Hyperarousal Index
CRI	Cortical Reactivity Index
PSD	Power Spectral Density
DSM-5	Diagnostic and Statistical Manual of Mental Disorders, Fifth Edition
MNE	Magnetoencephalography and Electroencephalography (MNE-Python analysis framework)
EDF	European Data Format
IFCN	International Federation of Clinical Neurophysiology
ADHD	Attention-Deficit/Hyperactivity Disorder
